# Aging, cardiotoxicity, and chemotherapy

**DOI:** 10.18632/aging.101776

**Published:** 2019-01-22

**Authors:** Edimar Alcides Bocchi, Mônica Samuel Avila, Silvia Moreira Ayub-Ferreira

**Affiliations:** 1 Heart Institute of São Paulo University Medical School, São Paulo, Brazil

**Keywords:** cancer, cardiomyopathy, heart failure, chemotherapy, cardiotoxicity, carvedilol

Recently, the Carvedilol Effect in Preventing Chemotherapy-Induced Cardiotoxicity (CECCY) trial showed that carvedilol had a neutral effect on the primary prevention of anthracycline-induced cardiotoxicity evaluated by left ventricular ejection fraction (LVEF) after 6 months of follow-up (absolute reduction of 1.3% in placebo and 0.9% in carvedilol group, p=ns) [1]. However, carvedilol significantly decreased troponin levels and diastolic dysfunction. The impact of reduced troponin levels on long-term follow-up is unknown. Otherwise, troponin proliferation is associated with subclinical cardiac injury, and it is a predictor of cardiac death and the occurrence of heart failure. The CECCY trial is the largest randomized placebo-controlled trial for primary prevention of anthracycline induced-cardiotoxicity that has tested the efficacy of carvedilol. It included 200 breast cancer patients with a low risk of cardiovascular disease who were receiving carvedilol at a cumulative dose of anthracycline of 240 mg/m^2^.

Chemotherapy-induced cardiotoxicity is epidemiologically relevant based on data showing that the number of cancer survivors continues to increase because of aging, better access to health care systems with early detection and treatment, palliative care, and advances in treatment methods. It is estimated that more than 20 million Americans with a history of cancer will be alive on January 1, 2026 [2]. Anthracyclines are used extensively to treat lymphoma, sarcoma, breast cancer, and pediatric leukemia. Chemotherapy based on anthracycline compounds may cause dose-dependent cardiotoxicity putting the success of treatment as risk. Recently, in long-term childhood cancer survivors, systolic dysfunction was detected in 5.8%, and abnormal global longitudinal strain and diastolic dysfunction was detected in 28% and 8.7%, respectively [3]. Heart failure (HF) incidences of 5%, 16%, and 26% were estimated for cumulative doxorubicin doses of 400, 500, and 550 mg/m^2^, respectively [4].

Mechanisms of anthracycline-induced cardiotoxicity include the generation of excess reactive oxygen species and formation of iron complexes. Recently, it was revealed that anthracyclines can inhibit topoisomerase 2β causing double-stranded breaks in DNA, which can lead to cardiomyocyte death [3]. Risk factors for anthracycline-related cardiotoxicity include the lifetime cumulative dose, infusion regimen, pre-existing cardiac disease, and cardiovascular risk factors, such as hypertension, diabetes, dyslipidemia, obesity, and older age (> 65 years) [5]. The increase in the incidence cancers of the breast (female) and colon-rectum and non-Hodgkin lymphoma as age increases indicates the aging process as a risk marker for greater development of cardiotoxicity in the population [2].

Strategies have been proposed for cardiotoxicity prevention, such as reduction in the cumulative anthracycline dose, continuous infusion, liposomal forms of the drugs, and the use of cardioprotective agents. Dexrazoxane is the only FDA-approved cardioprotective drug for prevention. Among cardiovascular drugs, the literature recommendations for cardiotoxicity are controversial. The hypothesis that carvedilol would prevent cardiotoxicity is supported by its antioxidant and antiapoptotic proprieties, and preservation of mitochondrial function. However, the primary prevention of cardiotoxicity with carvedilol until recently was based on trials with design limitations and the use of elevated anthracycline doses.

It is understood that the risk of cardiotoxicity with conventional anthracyclines increases in older age independent of comorbidities and performance status. Also, older patients have greater cardiovascular risk factors [6]. Despite the importance, older patients are generally under-represented in major clinical trials. In this scenario of poor scientific evidence, the potential benefits of treating the tumor should be balanced against the risk of worsening comorbidities [6]. However, age in itself should not prevent access to potentially curative treatment or treatment that increases longevity or improves quality of life.

In conclusion, cardiotoxicity seems to be an important potential burden to health systems, including in the older population. However, the CECCY trial showed that in patients without risk factors, the incidence in cardiotoxicity may be lower than expected [1]. Improving prevention and controlling risk factors with an individualized approach may be the best way to avoid cardiotoxicity in any age. This should include the balance between risks and benefits of chemotherapeutic treatment, with close monitoring of cardiac function and biomarkers during treatment [7,8]. The fact that cumulative and irreversible cardiotoxicity is likely to be greater in older than in younger patients calls for effective pretreatment screening for risk factors, rigorous monitoring of cardiac function, and early intervention [6]. New trials concerning prevention of cardiotoxicity in older populations are required.
